# Outcomes of Clinical Trials on Osteonecrosis of the Jaw

**DOI:** 10.7759/cureus.17984

**Published:** 2021-09-15

**Authors:** Brendan W Wu, Kevin C Lee, Steven Halepas, Vasiliki Karlis

**Affiliations:** 1 Oral and Maxillofacial Surgery, New York University Langone Health, New York, USA; 2 Oral and Maxillofacial Surgery, NewYork-Presbyterian/Columbia University Irving Medical Center, New York, USA; 3 Oral and Maxillofacial Surgery, NYC Health + Hospitals/Bellevue, New York, USA

**Keywords:** medication-related osteonecrosis of the jaw, bisphosphonate-related osteonecrosis of the jaw, osteoradionecrosis of the jaw, clinical trials, mronj, bronj, ornj, nct

## Abstract

Objective: The purpose of this study was to provide a cross-sectional view of all registered clinical trials enrolling patients with osteonecrosis of the jaw (ONJ). The primary aim was to report predictors of trial completion and publication of results.

Materials and Methods: This is a cross-sectional study of ONJ trials registered with ClinicalTrials.gov. For each included entry, trial characteristics and endpoints were recorded. Predictors were enrollment size, etiology, study type, intervention type, sponsor, funding, study locations, number of centers, and specialty of the principal investigator. Outcomes were trial status, publication on PubMed, journal of publication, and length of time between endpoints. Associations between predictors and outcomes were evaluated using chi-square tests and t-tests.

Results: The final sample included 26 trials. Overall, 50% of trials were completed and 69% of completed trials were published. Three out of four terminated trials were suspended due to lack of funding. The median enrollment for completed trials was 149 participants with a mean length of five years. All trials included medication-related osteonecrosis of the jaw (MRONJ) patients and 26% also included osteoradionecrosis of the jaw (ORNJ) patients. The majority of trials were observational (65%), conducted internationally (62%), and involved multiple centers (54%). Published trials had a mean time of 5.9 years between trial start and publication, which was comparable to trial length (p=0.90) and appeared in either dental (44%) or cancer (56%) journals. Completion and publication rates were not significantly increased by industry sponsorship/funding, larger enrollment sizes, or multi-center involvement. Oral and maxillofacial surgery was the most represented dental specialty of principal investigators (56%).

Conclusions: The majority of completed ONJ trials had their results published in a timely manner. Evidence-based investigation of ONJ is a multi-disciplinary and international effort. Among all specialists, oral and maxillofacial surgeons led the most ONJ trials.

## Introduction

The goal of evidence-based healthcare is to make clinical decisions based on objective data. Ideally, all clinical decisions would be supported by level I evidence, which requires the presence of at least one randomized controlled trial. Unfortunately, up to half of all randomized controlled trials do not result in a publication. Unpublished research hinders medical advancement. Discoveries should be reported regardless of the results [1], and publication bias may lead to false and inappropriate conclusions [2]. Unpublished or incomplete clinical trials also waste research dollars that could have otherwise been allocated to other more fruitful efforts. Although the randomized controlled trial is the most rigorous form of research, there are many barriers that preclude its success. 

Osteonecrosis of the jaws (ONJ) research is relatively young in comparison to other medical diagnoses. ONJ is of great research interest because there are few consensuses regarding its medical and surgical treatment. ONJ has been described in the literature dating back to over a century, and it has a variety of etiologic subtypes such as osteoradionecrosis of the jaw (ORNJ) from radiation. The recent interest in ONJ research was spurred by an increasing number of cases attributed to the use of anti-resorptive medications.

Bisphosphonate-related osteonecrosis of the jaw (BRONJ) was first reported by Marx in 2003 [3], and a variety of well-described risk factors have since been identified [4]. In 2014, the American Association of Oral and Maxillofacial Surgeons (AAOMS) renamed BRONJ as medication-related osteonecrosis of the jaw (MRONJ) in order to respect the role of other medications (e.g. receptor activator of nuclear factor kappa-Β ligand (RANK-L) inhibitors, anti-angiogenic agents, mechanistic target of rapamycin (mTOR) inhibitors) in the disease process [5]. The resurgence of interest in ONJ has led to an increase in the number of literature publications. Clinical trials investigating ONJ are of great importance to oral and maxillofacial surgery (OMS) because many patients with ONJ seek OMS care. The aim of this study is to identify factors associated with the successful completion and/or publication of ONJ trials.

## Materials and methods

A cross-sectional study was conducted using ClinicalTrials.gov, a publicly available clinical trial registry run by the United States (US) National Library of Medicine at the National Institutes of Health (NIH) [6]. The database was searched for entries containing the key phrases “jaw osteonecrosis,” “osteonecrosis of jaw,” and “osteonecrosis of the jaw”. The National Clinical Trial (NCT) number and trial characteristics were recorded.

Predictor variables included actual or estimated enrollment size, etiology (MRONJ or ORNJ), study type (observational or interventional), intervention type (drug, procedure, or biological) for interventional studies, trial sponsor (industry, research institute, university, or medical center), source of funding (industry, NIH, or other), location of the trial (US or international), number of centers (single or multi-center), the specialty of the principal investigator (oral and maxillofacial surgery, pharmacology, otolaryngology, oral pathology, etc.), trial start date, and trial completion date if applicable. Outcome variables were trial status (recruiting, completed, withdrawn, or unknown), and for completed trials, trial length, publication of results on PubMed (yes or no), journal of publication, publication date, and publication length (time between the start of the trial and the publication of results).

For trials with a status of “unknown” or “withdrawn”, the designated contact or principal investigator was emailed to ascertain the reasons behind the status. For completed trials, PubMed was queried with the principal investigator’s name and the keyword “osteonecrosis”. Putative articles were searched for the NCT number in the methods section to confirm association with the trial. When publication on PubMed could not be identified, the trial contact or principal investigator were emailed to inquire about the status of results publication. 

Descriptive statistics were calculated for all study variables. Associations between predictor and outcome variables were determined using chi-square tests, Fisher’s exact tests, and independent- or paired-samples t-tests. A p < 0.05 was considered statistically significant. Effect sizes were reported as odds ratio (OR) for categorical variables. Data were reported as mean ± standard deviation. Analyses were performed using Statistical Analysis System (SAS) software, Version 9.4 (released 2013, SAS Institute Inc., Cary, North Carolina) and IBM SPSS Statistics for Windows, Version 26.0 (released 2019, IBM, Armonk, New York). As per New York University Langone Health policy, research involving the analysis of de-identified data from publicly available datasets does not require institutional research board approval.

## Results

The final sample included 26 clinical trials pertaining to osteonecrosis of the jaw (Table 1). At the time of this study, 13 (50%) trials were completed while seven (27%) were still recruiting. Of the three trials with unknown status on ClinicalTrials.gov, two were clarified by principal investigators as withdrawn due to lack of funding (NCT02218554, NCT02566681) and one as still active (NCT02198001). One trial (NCT02069340) was withdrawn due to a lack of funding and enrollment. Actual enrollments ranged from 0 to 572,606 with a median of 149 participants. Estimated enrollments for incomplete trials ranged from 10 to 490 with a median of 120 participants. Actual and estimated enrollment sizes were not significantly different (p = 0.40). All trials included MRONJ patients (26; 100%); 16 (62%) trials only included BRONJ patients and six (23%) trials also included ORNJ patients. Nine (35%) trials included denosumab and nine (35%) trials included anti-angiogenic agents as etiologies.

**Table 1:** Characteristics of clinical trials on osteonecrosis of the jaw Data sourced from ClinicalTrials.Gov [6]. NCT: National Clinical Trial; N/A: not applicable; NIH: National Institutes of Health; US: United States; OMS: oral and maxillofacial surgery

NCT Number	Status	Enrollment Size (n)		Study Type	Intervention Type	Sponsor	Funding	Location	Center	Specialty
NCT02932501	Recruiting	500	(Estimated)	Observational	N/A	Research Institute	Other	International	Multi	OMS
NCT01666106	Active, not recruiting	327	(Actual)	Observational	N/A	Industry	Industry	International	Multi	Pharmacology
NCT01130389	Completed	309	(Actual)	Observational	N/A	Medical Center, Research Institute	NIH, Other	US	Multi	Oral Health
NCT03040778	Recruiting	100	(Estimated)	Interventional	Drug: pentoxifylline, tocopherol, placebo	Medical Center, University	Other	US	Multi	OMS
NCT01967160	Completed	2560	(Actual)	Observational	N/A	Medical Center, Industry	Industry, Other	International	Multi	Pharmacology
NCT01325142	Completed	271	(Actual)	Observational	N/A	Medical Center, University	NIH, Other	US	Multi	Breast Cancer
NCT01201330	Completed	572,606	(Actual)	Observational	N/A	Medical Center, Research Institute	NIH, Other	US	Multi	Oral Health
NCT03418454	Recruiting	150	(Estimated)	Observational	N/A	Medical Center	Other	International	Single	Oral Pathology
NCT02198001	Unknown	100	(Estimated)	Interventional	Biological: platelet rich fibrin	University	Other	International	Single	OMS
NCT02218554	Unknown	150	(Estimated)	Interventional	Procedure: genetic assay	Industry	Industry	International	Single	Oral Medicine
NCT02069340	Withdrawn	0	(Actual)	Interventional	Drug: zoledronic acid	Research Institute, University	NIH, Other	US	Multi	Oral Pathology
NCT03390777	Not yet recruiting	150	(Estimated)	Interventional	Biological: plasma rich in growth factors	University	Other	International	Multi	OMS
NCT00858585	Completed	149	(Actual)	Observational	N/A	Medical Center, University	Other	US	Single	Breast Cancer
NCT01998607	Completed	484	(Actual)	Observational	N/A	Industry	Industry	International	Multi	Pharmacology
NCT00874211	Completed	3571	(Actual)	Observational	N/A	Industry, Medical Center, Research Institute	Industry, NIH, Other	International	Multi	Breast Cancer
NCT00601068	Completed	35	(Actual)	Observational	N/A	Industry, University	Industry, Other	US	Single	Oral Pathology
NCT01875458	Recruiting	500	(Estimated)	Observational	N/A	University	Other	US	Single	OMS
NCT04012320	Completed	99	(Actual)	Observational	N/A	Medical Center	Other	International	Single	Pediatrics
NCT00462098	Completed	54	(Actual)	Interventional	Drug: hyperbaric oxygen	University	Other	US	Single	Anesthesiology
NCT02566681	Unknown	10	(Estimated)	Interventional	Biological: marrow stem cell construct	Medical Center, Research Institute	Industry, Other	International	Single	OMS
NCT04257721	Recruiting	120	(Estimated)	Observational	N/A	Medical Center	Other	International	Multi	OMS
NCT03269214	Completed	20	(Actual)	Interventional	Drug: topical phenytoin	University	Other	International	Single	OMS
NCT00592982	Completed	25	(Actual)	Observational	N/A	University	Other	US	Single	ENT
NCT00434447	Completed	73	(Actual)	Interventional	Drug: zoledronic acid	Industry	Industry	International	Multi	Pharmacology
NCT04007783	Recruiting	80	(Estimated)	Observational	N/A	Medical Center	Other	International	Multi	Prosthodontics
NCT02661139	Recruiting	55	(Estimated)	Observational	N/A	Medical Center	Other	International	Single	OMS

There were 17 (65%) observational studies and the remaining nine (35%) were interventional. Observational studies were not more likely to be completed than interventional studies (p = 0.22). The most common intervention was the treatment of osteonecrosis with a drug (56%). Trials were sponsored by medical centers (13; 50%), industry (7; 27%), research institutes (6; 23%), or universities (11; 42%). Funding was provided by the NIH (5; 19%), industry (8; 31%), or another source (22; 85%). Trial completion rates were higher with sponsorship from industries (71% vs. 42%), medical centers (54% vs. 46%), and universities (55% vs. 47%), as well as with funding from industry (63% vs. 44%) and the NIH (80% vs. 43%). The majority of trials had locations outside the US (16; 62%) and involved multiple centers (14; 54%). The specialty of the principal investigator was usually in a dental-related field (16; 62%), with oral and maxillofacial surgery most highly represented (9; 35%).

Characteristics of the 13 completed trials were investigated (Table 2). The mean trial length was 5.0 ± 3.3 years. Interventional trials were not longer than observational trials (p = 0.43), and multi-center trials were not longer than single-center trials (p = 0.64). Nine (69%) trials were published on PubMed with a mean time of 5.9 ± 2.5 years between trial start and publication. Publication length was not significantly longer than trial length (p = 0.90), and larger enrollments did not increase publication likelihood (p = 0.52). Published studies had a trial length of 5.8 ± 3.5 years whereas unpublished studies had a trial length of 3.1 ± 2.0 years (p = 0.19). Publication rates were higher for single-center trials (86% vs. 50%, OR = 6.0) and for trials with industry sponsorship/funding (80% vs. 63%, OR = 2.4) or research institute sponsorship (100% vs. 60%, OR = 2.0) (Table 3). Publications were either in cancer journals (5; 56%) or dental journals (4; 44%).

**Table 2:** Characteristics of completed clinical trials on osteonecrosis of the jaw Data sourced from ClinicalTrials.Gov [6]. NCT: National Clinical Trial; N/A: not applicable

NCT Number	Start Date	Completion Date	Trial Length (years)	Publication on PubMed	Journal	Publication Date	Publication Length (years)
NCT01130389	1/1/07	9/1/08	1.67	Yes [7]	J Dent Res	2/11/11	4.11
NCT01967160	1/2/12	8/5/19	7.59	Yes [8]	Support Care Cancer	12/23/17	5.98
NCT01325142	8/1/10	11/1/14	4.25	No	N/A	N/A	N/A
NCT01201330	1/1/07	5/1/08	1.33	Yes [9]	J Dent Res	2/11/11	4.11
NCT00858585	3/1/09	9/1/16	7.50	Yes [10]	Mol Oncol	7/29/15	6.41
NCT01998607	2/4/13	5/15/15	2.28	Yes [11]	Support Care Cancer	6/18/14	1.37
NCT00874211	12/1/08	6/1/19	10.50	Yes [12]	Support Care Cancer	12/7/16	8.02
NCT00601068	12/1/07	7/1/11	3.58	No	N/A	N/A	N/A
NCT04012320	10/31/18	12/31/18	0.17	No	N/A	N/A	N/A
NCT00462098	8/1/06	12/1/10	4.33	Yes [13]	J Oral Maxillofac Surg	6/12/12	5.86
NCT03269214	9/1/12	3/30/17	4.58	No	N/A	N/A	N/A
NCT00592982	10/1/06	8/1/13	6.83	Yes [14]	J Oral Maxillofac Surg	9/25/13	6.98
NCT00434447	12/1/06	2/27/17	10.24	Yes [15]	Eur J Cancer Care	1/30/17	10.16

**Table 3:** Predictors of clinical trial completion and publication NIH: National Institutes of Health

	Completion	Publication
Study Type		
Interventional	33.3%	66.7%
Observational	58.8%	70.0%
Industry Sponsor		
Yes	71.4%	80.0%
No	42.1%	62.5%
Medical Center Sponsor		
Yes	53.8%	71.4%
No	46.2%	66.7%
Research Institute Sponsor		
Yes	50.0%	100.0%
No	50.0%	60.0%
University Sponsor		
Yes	54.5%	50.0%
No	46.7%	85.7%
Industry Funding		
Yes	62.5%	80.0%
No	44.4%	62.5%
NIH Funding		
Yes	80.0%	75.0%
No	42.9%	66.7%
Location		
National	70.0%	71.4%
International	37.5%	66.7%
Center		
Single	50.0%	85.7%
Multi	50.0%	50.0%

## Discussion

The 26 clinical trials on osteonecrosis of the jaw had a completion rate of 50%. All three withdrawn trials experienced a lack of funding. This reflects the high financial demand of running a clinical trial and the importance of solidifying a funding source before initiation [16]. Completed trials had a reasonable publication rate of 69% with one trial under review by a journal. This proportion was higher than the 46% [17], 48% [18], and 54% [19] publication rates of ClinicalTrials.gov trials in other areas of research. Results were published without significant delay because the lengths of time to trial completion and publication were comparable. Although trials securing industry funding and sponsorship had a higher likelihood of completion and publication, the boost was not significant and was consistent with other studies [17,19]. Completion and publication rates did not positively correlate with enrollment size or the number of participating centers. Therefore, investigators with smaller teams or limited connections should not be averse to conducting ONJ trials.

Clinical trials on ONJ were largely supervised by investigators in a dental-related field (62%) followed by pharmacology (15%). Oral and maxillofacial surgery was the most represented dental specialty (56%), perhaps owing to surgical treatment of ONJ primarily by oral and maxillofacial surgeons. Pharmacology was likely represented due to the clinical significance of MRONJ.

Publications were limited to cancer and dental journals. Cancer publications were prevalent, and this is unsurprising due to the comorbidities of many MRONJ patients treated with osteoclast inhibitor therapy [8]. One publication in the Journal of Dental Research on the risk factors of bisphosphonate-related ONJ was the 58th most-cited publication on MRONJ [8,20]. All trials included MRONJ patients, but less than one-fourth included ORNJ patients. MRONJ was only recently described by Marx [3], and this research focus likely reflects the perceived novelty of MRONJ relative to other forms of ONJ.

There were several limitations to this study. We were only able to identify a limited number of registered trials and this reduced our statistical power. It should be noted that not all trials need to be registered on ClinicalTrials.gov, such as international ones without US subjects, further reducing the sample size. Future studies should revisit ClinicalTrials.gov to monitor the progress of ongoing studies and see how the registry responds to the discovery of novel therapies.

## Conclusions

In conclusion, the majority of completed ONJ trials had their results published in a timely manner. Terminated trials cited a lack of funding or enrollment. Completion and publication rates were higher for trials with industry, medical center, and research institute sponsorship and for those with industry and NIH funding. MRONJ as an etiology was more widely studied than ORNJ, and all trials included BRONJ. Evidence-based clinical investigation of ONJ is a highly multi-disciplinary and international effort, calling forth the world’s experts in oral and maxillofacial surgery, pharmacology, oncology, otolaryngology, oral pathology, prosthodontics, and more.
